# Influence of Genetic Admixture Components on CYP3A5*3 Allele-Associated Hypertension in Amerindian Populations From Northwest Mexico

**DOI:** 10.3389/fphar.2020.00638

**Published:** 2020-05-11

**Authors:** Carlos Galaviz-Hernández, Blanca P. Lazalde-Ramos, Ismael Lares-Assef, Alejo Macías-Salas, Margarita A. Ortega-Chavez, Héctor Rangel-Villalobos, Martha Sosa-Macías

**Affiliations:** ^1^Academia de Genómica, CIIDIR-Durango, Instituto Politécnico Nacional, Durango, México; ^2^Unidad Académica de Ciencias Químicas, Universidad Autónoma de Zacatecas, Zacatecas, México; ^3^Patología, Instituto de Seguridad y Servicios Sociales de los Trabajadores del Estado, Durango, México; ^4^Instituto de Investigación en Genética Molecular, Centro Universitario de la Ciénega, Universidad de Guadalajara, (CUCiénega-UdeG), Ocotlán, México

**Keywords:** CYP3A5, polymorphisms, Amerindian, Mexican, hypertension, ancestry

## Abstract

*CYP3A5* metabolizes endogenous substrates and ~30% of prescription drugs. The *CYP3A5* gene contains an active CYP3A5*1 allele, and a non-functional version, the CYP3A5*3 (rs776746), with consequences for drug therapeutic responses and side effects. Both CYP3A5*1 and *3 have been associated with hypertension. The frequency of CYP3A5*3 varies between populations of different ancestries, with Europeans having the highest allele frequency (> 90%). Given the importance of CYP3A5*3 in drug response and hypertension development, the aim of the present study was to evaluate the frequency of this polymorphism and its association with hypertension in vulnerable indigenous populations in Mexico. A total of 372 subjects were recruited from eight ethnic groups in Northwest Mexico. Systolic (SBP), diastolic (DBP), and median (MBP) blood pressures as well as body mass index (BMI) were measured. Ancestry was evaluated through STR analysis, and the CYP3A5*1/*3 polymorphisms were identified using real-time PCR with TaqMan® probes. Higher frequencies of CYP3A5*1 and *3 were observed in groups with higher (>90%) and lower (<90%) Amerindian ancestry, respectively. The CYP3A5*3/*3 genotype was more frequent in indigenous women with higher SBP and DBP values. On the other hand, the *1 allele showed a protective effect against both high SBP (OR, 0.38; 95% CI, 0.17–0.83, *p* = 0.001) and DBP (OR 0.38, 95% CI 0.18–0.81, *p* = 0.007) in women. This association remained significant after adjusting for BMI and age for diastolic (OR, 0.38; 95% CI, 0.17–0.84, *p* = 0.011) and systolic BP (OR, 0.33; 95% CI, 0.15–0.76, *p* = 0.005) BP levels in women. Thus, the frequency of CYP3A5*3 varies between groups and seems to depend on ancestry, and CYP3A5*1 decreases the risk of hypertension in Mexican indigenous women. This population analysis of CYP3A5*1/*3 has profound implications not only for the susceptibility to diseases, such as hypertension, but also for safer drug administration regimens, assuring better therapeutic responses and fewer side effects.

## Introduction

The CYP3A5 enzyme is a member of the cytochrome P450 (CYP) 3A subfamily, which metabolizes ~30% of the drugs used in clinical practice (Zanger and Schwab, 2013). CYP3A5 protein is expressed mainly in the liver (Zhang et al., 2016) with an extrahepatic expression predominantly at the level of the renal proximal tubule (Givens et al., 2003; Bolbrinker et al., 2012). In kidney cells, CYP3A5 catalyzes the 6b-hydroxylation of corticosterone and cortisol (Grogan et al., 1990; Schuetz et al., 1992), increasing renal retention of Na^+^ and influencing blood pressure (Watlington et al., 1992; Ghosh et al., 1995). The renal expression of CYP3A5 is variable (Haehner et al., 1996) and depends mainly on a non-functional polymorphism in intron 3 called CYP3A5*3 (6986A > G, rs776746), which causes RNA splicing, resulting in protein termination at amino acid 109 (Kuehl et al., 2001). The frequency of CYP3A5*3 varies considerably across populations, with the highest frequencies in Europeans (94%) and admixed Americans (80%), and the lowest in Africans (18%) (Zhou et al., 2017). Thompson et al. (2004) reported that the frequency of CYP3A5*3 shows an unusual geographic distribution and increases significantly with distance from the equator. This could be because the functional reference allele CYP3A5*1 may confer a selective advantage in dry weather by increasing Na^+^ and water retention (Kuehl et al., 2001).

In several studies, CYP3A5 has been associated with hypertension in humans, although the results have been controversial, as evidenced by the review described by Bochud et al. (2009) and other recent studies (Fisher et al., 2016; Li et al., 2017). Ethnicity of study subjects may help explain these inconsistencies, as it has been demonstrated that the association of the CYP3A5*1 allele with higher blood pressure occurs mainly in individuals of African descent, while in Caucasians it has only been observed in older individuals.

In Mexico, seven million inhabitants speak an indigenous language. In the northwest of the country, the main ethnic groups are distributed in two well-defined geographical regions. Five indigenous groups, the Coras, Huicholes, Tepehuanos, Tarahumaras, and Mexicaneros inhabit the Sierra Madre Occidental where the mean annual temperature is 19°C. On the other hand, the Seris, Guarijíos, and Mayos are located in semi-desert regions with a mean annual temperature of 30°C.

Currently, the distribution of the CYP3A5*3 polymorphism in Mexican indigenous groups is unknown, and the association of the *CYP3A5* gene with hypertension has not been reported. Thus, the aim of the present study was to determine the frequency of *CYP3A5* polymorphisms, and their association with hypertension in Mexican Amerindians.

## Materials and Methods

### Subjects

A total of 372 unrelated volunteers belonging to 8 different indigenous ethnicities of Northwest Mexico were studied. The sample included 94 Tepehuanos, 62 Huicholes, and 34 Mexicaneros from the state of Durango, 66 Tarahumaras from the state of Chihuahua, 58 Coras from the state of Nayarit and 14 Seris, 14 Guarijíos, and 30 Mayos from the state of Sonora. The study protocol was approved by the Ethics and Research Committee of the Durango General Hospital of the Mexican Health Ministry (Number 031/007). All subjects signed an authorized informed consent form after being informed of the nature of the study, in accordance with the Declaration of Helsinki.

Participants were recruited from their respective communities between 2010 and 2013. All individuals self-reported as Amerindians, and their ancestry was confirmed by analyzing 15 short tandem repeat (STR) loci (Sosa-Macías et al., 2013). Based on the results, the population was divided into high (HAA, > 90%) and low (LAA, < 90%) Amerindian ancestry. Medical histories and physical examinations were obtained from adult men and non-pregnant women to confirm that they were healthy. Volunteers diagnosed with diabetes, hypertension (HT), or undergoing anti-hypertensive treatment were excluded.

### Measurements

Height and weight were measured in the standing position without shoes using a standard stadimeter. Body mass index (BMI) was calculated as weight (kg) divided by height (m²). Overweight participants were classified based on a BMI ≥ 25 kg/m^2^.

Blood pressure (BP) was measured using a mercurial sphygmomanometer in triplicate to obtain the mean value as the final BP after the participant had been sitting for at least 5 min. A diagnosis of HT was defined as systolic blood pressure (SBP) ≥ 140 mm Hg, diastolic blood pressure (DBP) ≥ 90 mm Hg, and median arterial pressure (MAP) ≥ 105 mm Hg, based on the Seventh Report of the Joint National Committee on Prevention, Detection, Evaluation, and Treatment of High Blood Pressure (Jones and Hall, 2004).

### Admixture Analysis

The analysis of 15 STRs was performed using the AmpFISTR^®^ Identifiler Kit (Applied Biosystems). The amplified PCR products were analyzed using capillary electrophoresis in an ABI PRISM^®^ 3130 Genetic Analyzer, and genotypes were obtained using allelic ladders provided by the kit and the GeneMapper^®^ software 3.1 (Applied Biosystems).

### CYP3A5 Genotyping

A total of 5 mL of peripheral blood was drawn from an antecubital vein into a tube with EDTA and kept on ice during transportation to the laboratory. Genomic DNA was extracted using the QIAGEN Blood DNA Isolation Kit (QIAGEN, Hilden, Germany), and evaluated for integrity and concentration through 1% agarose electrophoresis and spectrophotometry, respectively. Genotyping was performed with quantitative real-time PCR using a TaqMan^®^ assay in a StepOne equipment (Applied Biosystems, Carlsbad, CA, USA). PCR amplification was performed in a 20 mL final volume containing 20 ng of template DNA, 1X TaqMan^®^ Genotyping Master Mix (Applied Biosystems), 1X specific TaqMan^®^ probe, and water. Thermal cycling conditions were as follows: an initial denaturation step of 10 min at 95°C, followed by 40 cycles of denaturation at 92°C for 15 s and annealing at 60°C for 1 min. Genotype identification was carried out using allelic discrimination software (Applied Biosystems). The TaqMan^®^ probe used to recognize CYP3A5*3 (rs776746) was C_26201809_30. Genotypes were evaluated in duplicate, and the results were confirmed through Sanger sequencing in 40 randomized samples (~10% of the total population).

### Statistical Analyses

Anthropometric parameters in the Mexican-Amerindian populations are presented as mean ± standard deviation, and comparisons were made using the Mann–Whitney *U* test. The inter-ethnic *CYP3A5* allele and genotype frequencies were compared using the χ^2^ and Fisher’s exact tests. The *CYP3A5* allele and genotype frequencies between normotensive and hypertensive subjects were performed using the Mann–Whitney *U* and Pearson’s χ^2^ tests. Statistical analyses were carried out using the statistical package SPSS^®^ version 25 for Windows (SPSS Inc., Chicago IL). Hardy–Weinberg equilibrium (HWE) was calculated using the χ^2^ goodness-of-fit test. The association between polymorphisms and HT was determined using multivariate logistic regression analysis, and the model was adjusted for age and BMI. These analyses were carried out using the SNPStats program (Solé et al., 2006). Statistical significance was established with a 95% confidence interval (CI) and a *p* value < 0.05.

## Results

A total of 372 Amerindian volunteers were enrolled, 120 (32.3%) men and 252 (67.7%) women. The anthropometric parameters for all populations investigated are summarized in **Table 1**. There were significant differences in BMI, SBP, DBP, and MAP between populations, which were higher in the Seris, Guarijios, and Mayos than in the other groups.

**Table 1:** Anthropometric parameters among Mexican-Amerindian populations.

	Seris, n = 14	Guarijíos, n = 14	Mayos, n = 30	Tarahumaras, n = 66	Mexicaneros, n = 34	Huicholes, n = 62	Coras, n = 58	Tepehuanos, n = 94
Age (years)	55.9 ± 13.9	58.1 ± 14.5	45.1 ± 18.2	42.8 ± 13.2	41.5 ± 13.9	40.1 ± 19.0	47.4 ± 20.9	36.6 ± 14.6
BMI (kg/m^2^)	27.3 ± 4.9*	27.6 ± 9.8*	27.8 ± 6.7*	24.1 ± 4.7	24.4 ± 3.9	23.5 ± 4.7	25.7 ± 5.3	22.3 ± 5.2
Systolic BP (mm Hg)	142.1 ± 27.8*	145.6 ± 19.6*	128.8 ± 20.1*	122.3 ± 19.0	117.1 ± 16.2	112.5 ± 21.2	120.6 ± 15.3	105.1 ± 16.4
Diastolic BP (mm Hg)	92.1 ± 12.5*	92.1 ± 7.8*	83.6 ± 11.7*	78.9 ± 9.4	74.7 ± 10.2	71.6 ± 12.4	78.4 ± 11.0	68.9 ± 11.6
MAP (mm Hg)	108.8 ± 16.4*	110.0 ± 10.4*	98.7 ± 14.0*	93.4 ± 12.0	88.8 ± 11.7	85.2 ± 14.6	92.5 ± 11.8	81 ± 12.8

Data are expressed as mean ± standard deviation.

*Mann–Whitney U test.

MAP, median arterial pressure; BMI, body mass index; BP, blood pressure.

Previously, we studied 15 STRs to estimate non-Amerindian ancestry in all the Native American populations evaluated here (Sosa-Macías et al., 2013). The highest European component was observed in the Mayo group, while the Tepehuano group had the highest indigenous component (**Table 2**). The distribution of *CYP3A5* genotypes deviates from HWE only in the Tepehuano group. The wild-type allele CYP3A5*1 was most frequent in the groups with higher indigenous ancestry: Mexicaneros (57.4%), Coras (33.6%), Tepehuanos (28.7%), Tarahumaras (28%), and Huicholes (11.3%). The highest frequencies of CYP3A5*3 were observed in the Mayos (96.7%), Seris (96.4%), and Guarijíos (92.9%) groups with higher European admixture. In these groups, no homozygote status (CYP3A5*1/*1) was detected, and the *1/*3 genotype frequency was lower than in groups with higher indigenous components. Higher homozygosity for the allele CYP3A5*3 was found in the Mayos (93.3%), Seris (92.9%), Guarijíos (85.7%), and Huicholes (80.6%).

**Table 2:** CYP3A5*3 allele and genotypic frequencies among Mexican-Amerindian populations.

Population	N			CYP3A5*3					
		Ancestry (%)^1^		Allele frequencies (%)		Genotypic frequencies (%)			HWE
		Amerindian	European	*1 (A)	*3 (G)	*1/*1 (A/A)	*1/*3 (G/A)	*3/*3 (G/G)	*p value^¤^*
Tepehuanos	94	96.4	3.1	28.7	71.3	12.8	31.9	55.3	0.04
Huicholes	62	96.3	3.1	11.3	88.7	3.2	16.1	80.6	0.16
Mexicaneros	34	94.5	4.3	57.4	42.6	38.2	38.2	23.5	0.29
Coras	58	93.9	4.7	33.6	66.4	15.5	36.2	48.3	0.15
Tarahumaras	66	92.1	7.0	28.0	72.0	10.6	34.8	54.5	0.36
Seris	14	88.0	11.0	3.6	96.4	0	7.14	92.9	1.00
Guarijíos	14	81.6	16.8	7.1	92.9	0	14.3	85.7	1.00
Mayos	30	65.6	32.6	3.3	96.7	0	6.7	93.3	1.00

HWE, Hardy–Weinberg equilibrium; ^¤^χ^2^ goodness-of-fit statistic. ^1^Sosa-Macías et al., 2013.

In order to determine relationships between blood pressure levels and CYP3A5*3 allele and genotypic frequencies, an analysis of the total population was carried out (**Table 3**). The frequency of allele *3 was significantly higher (84%) in Amerindians with diastolic BPs ≥ 90 mm Hg (*p* < 0.001). The *1/*3 genotype was the most frequent in subjects with diastolic BPs <90 mm Hg (29.8%), but only occurred in 17.8% of subjects with diastolic BPs ≥90 mm Hg (*p* = 0.04). A higher percentage of subjects with genotype *3/*3 was observed in the groups with the highest diastolic (75.3%) and systolic (74.6%) BP figures (*p* values 0.01 and 0.02, respectively). There were no significant differences in the allele and genotype frequencies of CYP3A5*3 in terms of MAP values.

**Table 3:** CYP3A5*3 allele and genotype frequencies in normotensive and hypertensive Mexican-Amerindian population.

	Diastolic BP, mm Hg		*p* value	Systolic BP, mm Hg		*p* value
	<90	≥90		<140	≥140	
	n=299	n=73		n=307	n=65	
BP	71.6 ± 9.5	94.6 ± 6.3	**0.000**	110.5 ± 14.2	151.8 ± 14.2	**0.000**
Age (years)	39.9 ± 15.6	53.9 ± 15.3	**0.000**	39.5 ± 15.4	57.4 ± 13.2	**0.000**
BMI (kg/m^2^)	23.8 ± 4.2	26.8 ± 5.6	**0.000**	24.1 ± 4.5	25.5 ± 5.5	**0.043**
Alleles						
*1 (A)	165 (27.6)	23 (15.8)	**0.002**	165 (26.9)	23 (17.7)	**0.029**
*3 (G)	433 (72.4)	123 (84.3)		449 (73.1)	107 (82.3)
Genotypes						
*1/*1 (A/A)	38 (12.7)	5 (6.8)	0.160	38 (12.4)	5 (7.7)	0.283
*1/*3 (A/G)	89 (29.8)	13 (17.8)	**0.040**	89 (29.1)	13 (20.0)	0.140
*3/*3 (G/G)	172 (57.5)	55 (75.3)	**0.005**	180 (58.6)	47 (72.3)	**0.040**

n (%). BP, blood pressure; BMI, body mass index.

Mann–Whitney U test, Pearson χ^2^, p < 0.005 in bold.

Gender analysis revealed significant differences only in Amerindian women (**Table 4**). The frequency of allele CYP3A5*3 was significantly higher in women with diastolic BPs ≥90 mm Hg and systolic BPs ≥140 mm Hg than in the groups with lower BP values (*p* = 0.01). In women with diastolic BPs <90 mm Hg the *1/*3 genotype was significantly more frequent (29.3%) than in females with diastolic BPs ≥90 mm Hg (14.9%) (*p* = 0.04). The *3/*3 genotype frequency was higher in the groups with higher diastolic (78.7%) and systolic (79.1%) BPs than in groups with lower figures (58.5% and 58.9% respectively, *p* = 0.01).

**Table 4:** CYP3A5*3 allele and genotype frequencies in normotensive and hypertensive women and men.

	Diastolic BP, mm Hg		*p* value	Systolic BP, mm Hg		*p* value
	<90	≥90		<140	≥140	
**Women**	n = 205	n = 47		n = 209	n = 43	
BP	71.4 ± 9.4	93.8 ± 5.6	**0.000**	109.7 ± 13.9	148 ± 9.2	**0.000**
Age (years)	38.8 ± 14.8	51.4 ± 15.2	**0.000**	37.9 ± 14.1	56.9 ± 13.1	**0.000**
BMI (kg/m^2^)	24.3 ± 4.3	27.5 ± 5.8	**0.000**	24.7 ± 4.5	25.7 ± 5.9	0.373
Alleles						
*1 (A)	110 (26.8)	13 (13.8)	**0.009**	112 (26.8)	11 (12.8)	**0.011**
*3 (G)	300 (73.2)	81 (86.2)		306 (73.2)	75 (87.2)
Genotypes						
*1/*1 (A/A)	25 (12.2)	3 (6.4)	0.253	26 (12.4)	2 (4.7)	0.185
*1/*3 (A/G)	60 (29.3)	7 (14.9)	**0.044**	60 (28.7)	7 (16.3)	0.093
*3/*3 (G/G)	120 (58.5)	37 (78.7)	**0.010**	123 (58.9)	34 (79.1)	**0.013**
**Men**	n=94	n=26		n=98	n=22	
BP	72 ± 9.7	96 ± 7.5	**0.000**	112.2 ± 14.8	159.3 ± 18.8	**0.000**
Age (years)	42.4 ± 17	58.5 ± 14.9	**0.000**	43.2 ± 17.4	58.4 ± 13.5	**0.000**
BMI (kg/m^2^)	22.7 ± 4	25.5 ± 5.1	**0.006**	22.9± 4.2	25.2 ± 4.5	**0.007**
Alleles						
*1 (A)	55 (29.3)	10 (19.2)	0.150	53 (27)	12 (27.3)	0.257
*3 (G)	133 (70.7)	42 (80.8)		143 (73)	32 (72.7)
Genotypes						
*1/*1 (A/A)	13 (13.8)	2 (7.7)	0.519	12 (12.24)	3 (13.6)	1.00
*1/*3 (A/G)	29 (30.9)	6 (23.1)	0.440	29 (29.6)	6 (27.3)	0.653
*3/*3 (G/G)	52 (55.3)	18 (69.2)	0.203	57 (58.2)	13 (59.1)	0.508

n (%). BP, blood pressure; BMI, body mass index.

Mann–Whitney U test, Pearson χ^2^. p < 0.05 in bold.

**Table 5** shows the results of the logistic regression analysis. After a crude and adjusted analysis under a dominant inheritance model, a significant negative association was found between CYP3A5*1 and high diastolic, systolic, and MAP values in the whole population. A similar association was also observed in the female groups, but not in terms of MAP values. The analysis of male groups did not reveal any association.

**Table 5:** Association of CYP3A5*1 and hypertension.

Population/variable	OR (95% CI)	*p* value
Whole cohort/DBP crude analysis	0.44 (0.25–0.79)	**0.004**
Whole cohort/DBP adjustment age, BMI	0.44 (0.23–0.83)	**0.009**
Whole cohort/SBP crude analysis	0.54 (0.30–0.98)	**0.036**
Whole cohort/SBP adjustment age, BMI	0.51 (0.27–0.97)	**0.034**
Whole cohort/MAP crude analysis	0.51 (0.27–0.96)	**0.029**
Whole cohort/MAP adjustment age, BMI	0.49 (0.24–0.97)	**0.033**
Women/DBP crude analysis	0.38 (0.18–0.81)	**0.007**
Women/DBP adjustment age/BMI	0.38 (0.17–0.84)	**0.011**
Women/SBP crude analysis	0.38 (0.17–0.83)	**0.001**
Women/SBP adjustment age/BMI	0.33 (0.15–0.76)	**0.005**
Women/MAP crude analysis	0.50 (0.23–1.12)	0.082
Women/MAP adjustment age/BMI	0.46 (0.19–1.11)	0.071
Men/DBP crude analysis	0.55 (0.22–1.39)	0.200
Men/DBP adjustment age/BMI	0.51 (0.18–1.43)	0.190
Men/SBP crude analysis	0.71 (0.26–1.94)	0.500
Men/SBP adjustment age/BMI	0.75 (0.25–2.27)	0.610
Men/MAP crude analysis	0.50 (0.18–1.40)	0.170
Men/MAP adjustment age/BMI	0.48 (0.16–1.47)	0.190

OR, odds ratio; DBP, diastolic blood pressure; SBP, systolic blood pressure;

MAP, median arterial pressure; BMI, body mass index. p < 0.05 in bold.

## Discussion

Our results show that the frequency of the CYP3A5*3 allele is higher in indigenous groups with lower Amerindian ancestry, while CYP3A5*1 decreases the risk of HT in Mexican indigenous women.

CYP3A5 metabolizes a great variety of drugs and endogenous compounds that regulate physiological processes including blood pressure. The expression of CYP3A5 depends in part on polymorphisms whose frequencies vary in different populations.

In the current study, the highest frequencies of CYP3A5*3 were observed in the three groups with the LAA (92.9 – 96.7%), which is similar to those reported for Europeans, Asians, and admixed Americans (> 90%) (Zhou et al., 2017). In the Tepehuano group exclusively, the observed genotype distributions deviated from HWE, likely resulting from the geographic isolation and endogamy present in the indigenous community from which most subjects were recruited. It is worth mentioning that the total number of participants from the different ethnic groups depended on both the number of inhabitants per community as well as the number of individuals who agreed to participate in the study.

On the other hand, the observed frequencies of CYP3A5*1 in the five groups with HAA and the groups with LAA ranged from 11.3% to 57.4% in the former, and 3% to 7% in the latter. **Table 6** shows the frequencies of CYP3A5*1 in each population. The frequencies in the groups with HAA coincide with those observed in Mexican Amerindians (30.3%) and Mestizos (18.9%) (Gonzalez-Covarrubias et al., 2019), and with those reported by Hustert et al. (2001) in three Asian populations (~30%). Meanwhile, the groups with LAA had similar frequencies as Caucasians (5%) (Hustert et al., 2001). Roy et al. (2005) evaluated Caucasian Canadians and reported frequencies of 7% for CYP3A5*1, an identical frequency to that found in our group with LAA. The frequency of CYP3A5*1 in French Caucasians, Gabonese, and Tunisian subjects was 0%, 5%, and 3%, respectively (Quaranta et al., 2006). In the Iranian population, the frequency of CYP3A5*1 was 8% (Azarpira et al., 2011), which is similar to that observed in Jordanians (7%) (Yousef et al., 2012). The low frequency in both populations was similar to that in the LAA groups in the present study. The evaluation of CYP3A5*1 in the Mexican Mestizo population revealed a frequency of 9% (Vargas-Alarcón et al., 2014), almost the same as that in the LAA Amerindian groups with the highest levels of European admixture (Seris, Guarijios, and Mayos).

**Table 6:** Comparison of CYP3A5 *1 frequency in different populations.

CYP3A5 *1 (rs776746)	Frequency (%)	Ref
Indigenous HAA	11.3 - 57	This study
Indigenous LAA	3 - 7	This study
Mexican Amerindian	30.3	Gonzalez-Covarrubias et al., 2019
Mexican Mestizo	18.9	Gonzalez-Covarrubias et al., 2019
MXL	23	Data from 1000 Genomes
CEU	4	Data from 1000 Genomes
YRI	83	Data from 1000 Genomes
CHB	31	Data from 1000 Genomes

HAA, high Amerindian ancestry; LAA, low Amerindian ancestry.

It is well known that the functional allele CYP3A5*1 is involved in sodium reabsorption and influences blood pressure (Eap et al., 2007). In 2003, Givens et al. demonstrated that the heterozygous genotype CYP3A5*1/*3 gave rise to a higher expression of the CYP3A5 enzyme in the kidneys compared to the inactive CYP3A5*3 homozygous form. The same study showed high systolic blood pressure values in African-American women with the CYP3A5*1/CYP3A5*1 genotype (Givens et al., 2003). Similar results have been found in other studies. In 2005, Ho et al. compared hypertensive vs. normotensive Caucasian and black subjects and found higher baseline DBP and SBP in the black group with CYP3A5*1/*1 or CYP3A5*1/*3 genotypes. The same results were observed after saline infusion and furosemide administration, but these results were not observed in white subjects (Ho et al., 2005). In African descendants, an age-dependent significant increase in BP values was observed only in those carrying the CYP3A5*1 allele (Bochud et al., 2006).

In Japanese men, the homozygous genotype *1/*1 was observed in subjects with high DBP, while no differences between genotypes were observed for SBP (Zhang et al., 2010). Conversely, in the present study the frequency of the homozygous genotype CYP3A5*1 was higher in normotensive subjects, although these results were not significant. A similar result was demonstrated in Caucasian women and men with low SBP values carrying the homozygous CYP3A5*1 genotype, although such an effect was not observed for DBP (Kreutz et al., 2005). In contrast, a German Caucasian population showed no association between high SBP or DBP and the CYP3A5*1 allele (Lieb et al., 2006).

On the other hand, in this study, the frequency of CYP3A5*1/*3 was significantly higher in women with lower DBP, while the CYP3A5*3/*3 genotype was over-represented in women with higher DBP and SBP. Such differences were not found in men.

The distributions of genotypes in the present study agree with those reported by Fromm et al. (2005), who found higher SBP in young Caucasian men with CYP3A5*3/*3, compared with subjects carrying CYP3A5*1/*3 genotype, suggesting a *3 allele dose-effect which could be associated with high systolic blood pressure (Fromm et al., 2005).

The functional allele CYP3A5*1 contributes to salt avidity (sodium retention) and hence HT. It is more frequent in populations closer to the equator and is considered an ancestral allele in African populations before diaspora (Thompson et al., 2004). We observed a protective effect of the *1 allele in women against high diastolic and systolic BP, which remained after adjusting for BMI and age [diastolic (OR 0.38, 95% CI 0.17–0.84, *p* = 0.011) and systolic BP (OR 0.33, 95% CI 0.15–0.76, *p* = 0.005) BP]. Conversely, no association was found in the male group, presumably because of the lower number of samples in this group (**Table 5**). In this study, we observed that populations with HAA showed the highest frequencies of the *1 allele and were the most normotensive groups. These populations are settled in mountainous communities with low environmental salt availability, so the presence of the *1 allele is beneficial, similar to what may have occurred in ancient populations in Africa. This phenomenon could explain the apparently paradoxical observation. On the other hand, the observed association in women could be the result of the higher number of women in this study.

Scarce reports exist about the prevalence of HT in indigenous Mexican populations. In 2008, Rodríguez-Moran et al. in the search for cardiovascular risk factors, evaluated two ethnic groups from Northwestern Mexico (Tepehuanos and Yaquis), who presented with HT in 3.3% and 6.3% of the study subjects, respectively (Rodríguez-Morán et al., 2008). The same authors showed in a follow up study an increase in the prevalence of HT from 1.7% in 1996 to 3.5% in 2006 in Tepehuano’s communities (Rodríguez-Morán et al., 2009). These data reveal that indigenous groups are adopting westernized habits, which is supported by the three groups in our study with LAA, who presented the highest values of BP and were located in more accessible and warmer communities with higher salt intakes. In this case, the presence of *3 was not sufficient to eliminate the high amount of salt, giving rise to the high BP values observed in these groups.

The distribution of *1 and *3 alleles in these populations and the influence of environmental factors, such as the use of traditional herbal remedies, can have repercussions in drug responses, making the evaluation of genetic profiles in indigenous groups more relevant.

This is the case in the Huichol group, which has the highest frequency of homozygous *3/*3 among the groups with the highest indigenous ancestry, which can be explained by the geographical differences between these groups. Huichols inhabit a region with little water availability, which could be an environmental pressure to maintain the *3 allele, promoting water retention without the development of hypertension. In addition, it is a community with little interaction with other groups, which has allowed them to maintain their habits and customs and could explain why they are among the groups with normal blood pressure.

Some limitations of this study deserve mention. Only one blood pressure measurement was performed. Because of the geographic isolation of these studied groups, a high rate of endogamy cannot be ignored. There were a high number of women compared to men. No measures of dietary salt content or urine salt elimination were performed. CYP3A5-interacting genes such as *AGT* were not evaluated.

To the best of our knowledge, this is the first study to evaluate the distribution of CYP3A5 *1 and *3 alleles and its association with HT in a Mexican indigenous population confirmed by molecular ancestry.

## Data Availability Statement

The raw data supporting the conclusions of this article will be made available by the authors, without undue reservation, to any qualified researcher.

## Ethics Statement

The studies involving human participants were reviewed and approved by Ethics and Research Committee of the Durango General Hospital of the Mexican Health Ministry (Number 031/007). The patients/participants provided their written informed consent to participate in this study.

## Author Contributions

CG-H: collection of samples, determination of polymorphisms, analysis of results, and wrote the paper. BL-R: collection of samples, biostatistical analyses. IL-A: preliminary analyses of results, critical review of manuscript. AM-S: determination of polymorphisms. MO-C: determination of polymorphisms. HR-V: determination of ancestry. MS-M: design of the study, collection of samples, final analysis of results, critical review of manuscript.

## Funding

This research was funded by grants from the Secretaría de Investigación y Posgrado (SIP) of Instituto Politécnico Nacional (SIP 20196043 and SIP20196451).

## Conflict of Interest

The authors declare that the research was conducted in the absence of any commercial or financial relationships that could be construed as a potential conflict of interest.
